# Walking alone milestone combined reading-frame rule improves early prediction of Duchenne muscular dystrophy

**DOI:** 10.3389/fped.2022.985878

**Published:** 2022-08-12

**Authors:** Yan-li Ma, Wei-hua Zhang, Guo-hong Chen, Li-fang Song, Yuan Wang, Rui-li Yuan, Ying Wang, Xiu-yong Cheng

**Affiliations:** ^1^Department of Neonatology, The First Affiliated Hospital of Zheng Zhou University, Zhengzhou, China; ^2^Department of Neurology, Children's Hospital Affiliated to Zhengzhou University, Henan Children's Hospital, Zhengzhou Children's Hospital, Zhengzhou, China; ^3^Department of Neurology, Beijing Children's Hospital, Beijing, China

**Keywords:** motor milestone, reading-frame, children, Duchenne muscular dystrophy, prediction

## Abstract

**Objective:**

To explore the potential of walking alone milestone combined reading-frame rule to improve the early diagnosis of Duchenne muscular dystrophy (DMD).

**Method:**

To retrospectively describe the genotype and phenotype of Duchenne and Becker muscular dystrophies (BMD) patients with deletions and duplicates in the dystrophin gene. The sensitivity and specificity of the reading frame rule were calculated and compared to that of the combined reading frame rule and walking alone milestone. The diagnostic coincidence rate of two different methods was analyzed.

**Result:**

One hundred sixty-nine male DMD/BMD patients were enrolled, including 17 cases of BMD and 152 cases of DMD. The diagnostic coincidence rate, diagnostic sensitivity, and specificity of the reading-frame rule for DMD/BMD were 85.2, 86.8, and 70.59%, respectively. The sensitivity and specificity of the reading frame principle combined with the walking alone milestone for DMD/BMD were 96.05 and 70.59%, respectively. The diagnostic coincidence rate increased to 93.49%, significantly different from that predicted by reading- frame rule (*P* < 0.05).

**Conclusion:**

The reading-frame rule combined with the walking alone milestone significantly improved the early diagnosis rate of DMD.

## Introduction

Dystrophinopathies, the most common type of progressive muscular dystrophy, is an X-linked recessive neuromuscular disorder caused by mutations in the dystrophin gene (*DMD*). The incidence rate did not vary significantly among countries, regions, or races, with one case occurring in every 3,600–6,000 male births (1).

*DMD* gene is located on chromosome Xp21.2, with a total length of 2.2 Mb and 79 exons, the largest gene discovered by humans so far. The majority of mutations in *DMD* gene are the deletion/duplication of one or more exons, accounting for about 70–80%. In addition, about 23% of the cases were caused by point mutations in the exon and flanking region of the gene (2).

Children with Duchenne muscular dystrophy (DMD) could not walk independently from 10-15 years old. Their average life expectancy under natural conditions was about 20 years (1). The phenotype of Becker muscular dystrophy (BMD) is relatively mild. BMD usually loses the ability to walk independently after 16 years old, with a life span of more than 30 years. Both DMD and BMD are due to mutations on the DMD gene, but the severity phenotype between the two forms varies considerably. At present, many gene therapy methods for DMD have been gradually applied in clinical practice, such as Ataluren for non-sense mutations in, exon 51 jump and other method, and early treatment can benefit more. Early prediction of DMD and BMD is needed to initiate treatment before the motor loss and joint contracture. “Reading-frame rule” is often used to distinguish BMD and DMD patients (3). Nevertheless, not all patients follow the “reading-frame rule,” and about 8% of DMD patients and 34% of BMD patients reportedly do not follow this rule (4).

Quantitative analysis of dystrophy protein in muscle biopsies can help identify DMD/BMD early, but invasive procedures limit its clinical application (5). An early developmental milestone is another variable to predict DMD. Dommelen and colleagues found delays in motor milestones in young males with DMD compared to the control group. Cyrulnik found that 70% of DMD children were delayed in walking alone milestones, followed by crawling (60%) and sitting (38%) (6). Previous studies have shown that gross motor milestones are potentially helpful for early diagnosis of DMD, especially at the age of first walking (6, 7).

This study aimed to evaluate the early predictive value of the reading-frame rule combined with the walking alone milestone for DMD.

## Materials and methods

### Data collection

A group of unrelated male probands from 208 cases diagnosed in Children's Hospital Affiliated to Zhengzhou University from 2014 to 2021. All the patients had confirmed met the criteria of DMD/BMD.

Inclusion criteria: (1) Male, (2) myopathy (elevated CK with or without proximal limb weakness, (3) genetic testing revealed *DMD* gene with a pathogenic variant.

Exclusion criteria: (1) Female, (2) the cases with point mutation in *DMD*, (3) at the last follow-up, the cases had not lost ambulation and were under 16.

Diagnostic principles of DMD and BMD: Patients who lose ambulation and need wheelchairs before 16 years old (<16 y) were classified as DMD, and patients who did not lose independent walking ability at 16 years old (≥16 y) were diagnosed as BMD.

We definite the “criteria for delay of walking alone milestone” as“unable to walk alone until 18 months old.”

**Figure 1:**
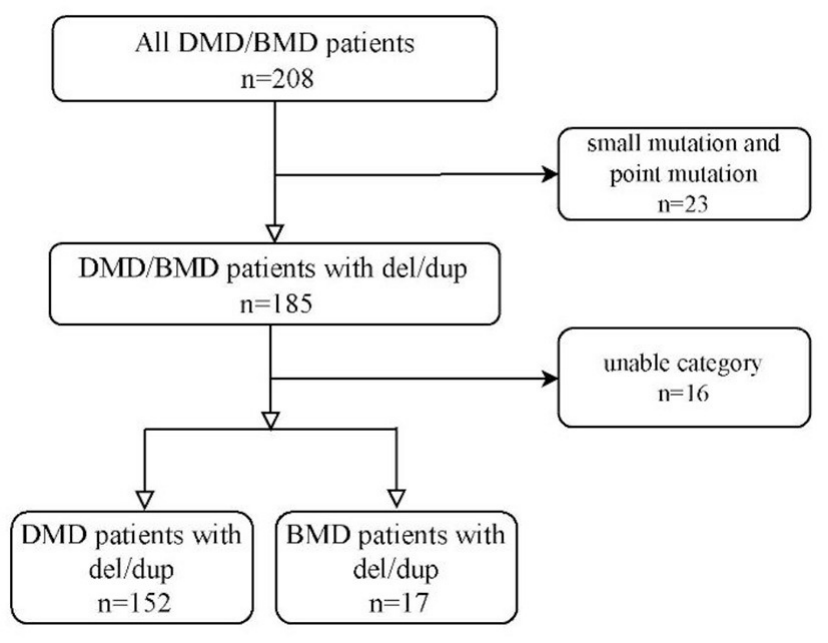
Study profile.

Two hundred eight cases were registered, 185 (88.9%) cases are deletions/duplications. Twenty-three (11.1%) cases were excluded because of the point mutation. Sixteen cases younger than 16 who could walk independently at the last follow-up were excluded. One hundred sixty-nine cases of DMD/BMD with deletion or duplication were included in our study, Figure 1. We retrospectively reviewed all cases' walking alone milestones and other demographic data.

### Statistical analysis

*T*-test was used to compare DMD and BMD's demographic and clinical data. The Chi-square test (χ^2^-test) was used to compare the diagnostic coincidence rate of different methods. Version 25 of the SPSS software (SPSS, Inc.) was used for all statistical analyses, setting the significance at *p* < 0.05.

### Ethical consent

The study was approved by the ethics committee of the Children's Hospital Affiliated to Zhengzhou University, Zhengzhou, China.

## Result

### Clinical findings

One hundred sixty-nine cases were included in our study. 20 cases had a family history, while the other 149 had no family history or family members refused to provide information. All 169 cases are male. The mean age of onset in DMD and BMD cases was 3.88 ± 2.18 years (range 0.5–9 years) and 6.49 ± 3.06 years (range 2.8–12 years), respectively, but the age of diagnosis delayed, which was 6.80 ± 2.83 (range 1.27–14.34 years) and 7.13 ± 3.45 (2.88 ± 12.12 years), respectively. The mean value of creatine kinase (CK) was 16,197.15 ± 8,750.95U/L (range 4,463–57,396 U/L) in DMD patients and 8,657.45 ± 5,933.32 U/L (range 1,199–20,048.6 U/L) in BMD patients, with the statistical difference (*P* < 0.05; Table 1). In DMD cases, the mean age of independent walking loss was 11.14 ± 1.07 years old (ranging from 8 to 14 years old), while in BMD cases, by our last follow-up time, all patients were older than 16 years old (maximum follow-up to 18 years old) and could walk independently.

**Table 1:** Clinical manifestations and investigations of DMD and BMD cases.

	**DMD (*****n*** = **152)**		**BMD (*****n*** = **17)**		***P*-value**
	**Mean ±SD**	**Min–Max**	**Mean ±SD**	**Min–Max**	
Walking alone milestone (month)	18.03 ± 7.12	14–30	12.88 ± 0.61	12–14	0.00
Onset age (year)	3.9 ± 2.18	0.5–9	6.49 ± 3.06	2.8–12	0.00
Age of diagnosis (year)	6.8 ± 2.83	1.27–14.34	7.16 ± 3.45	2.8–12	0.63
CK(U/L)	16,197.2 ± 8,750.9	4,463.0–57,296.0	8657.5 ± 5933.3	1,199.0–20,048.6	0.001

The age of walking alone in BMD group was between 12 and 14 months, with a mean age of 12.88 ± 0.61 months, while the age of walking alone in DMD group was between 14 and 30 months, with a mean age of 18.03 ± 7.12 months, Table 1. There were significant differences between the two groups (*P* < 0.05). In BMD group, all cases could walk independently before 18 months, while in DMD cases, 93 cases (61.18%) had an independent walking delay.

### Genotype

**Figure 2:**
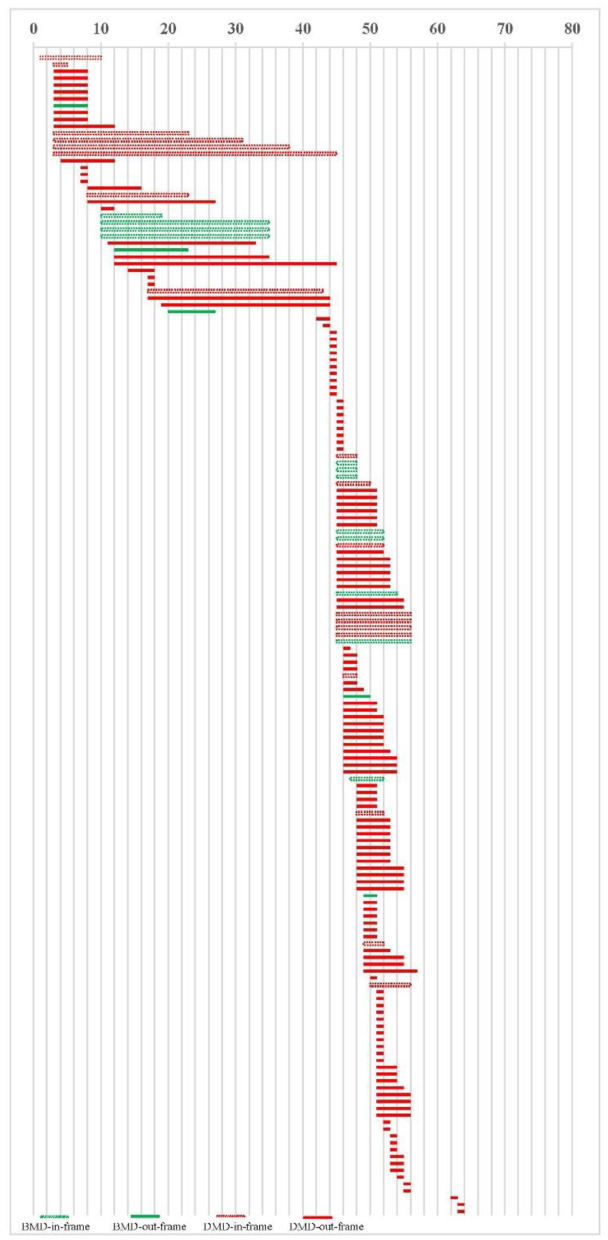
Deletion and duplication location in 169 cases.

Thirty-nine of the 169 cases had maternal genetic verification, including 8 with *de novo* mutations and 31 with maternal origin (Table 2). There were 153 cases of deletion, including 17 cases of BMD, 136 cases of DMD, and 16 cases of duplication, all of which were DMD. 17 cases are BMD, including 12 (70.6%) cases in the reading frame and 5 (29.4%) cases out of the reading frame. 152 cases are DMD, including 132 (86.8%) cases out of the reading frame and 20 (13.2%) cases in the reading frame, Figure 2.

**Table 2:** The genotype of 169 cases.

	Resource (M/D^*^)	del/dup^**^	Reading frame (in/out)^***^
BMD	1/1	17/0	12/5
DMD	30/7	136/16	20/132
Total	39	169	169

* *M, maternal; D, De novo*.

** *Del, deletion; dup, duplication*.

*** *In, in reading-frame; out, out of reading-frame*.

**Figure 3:**
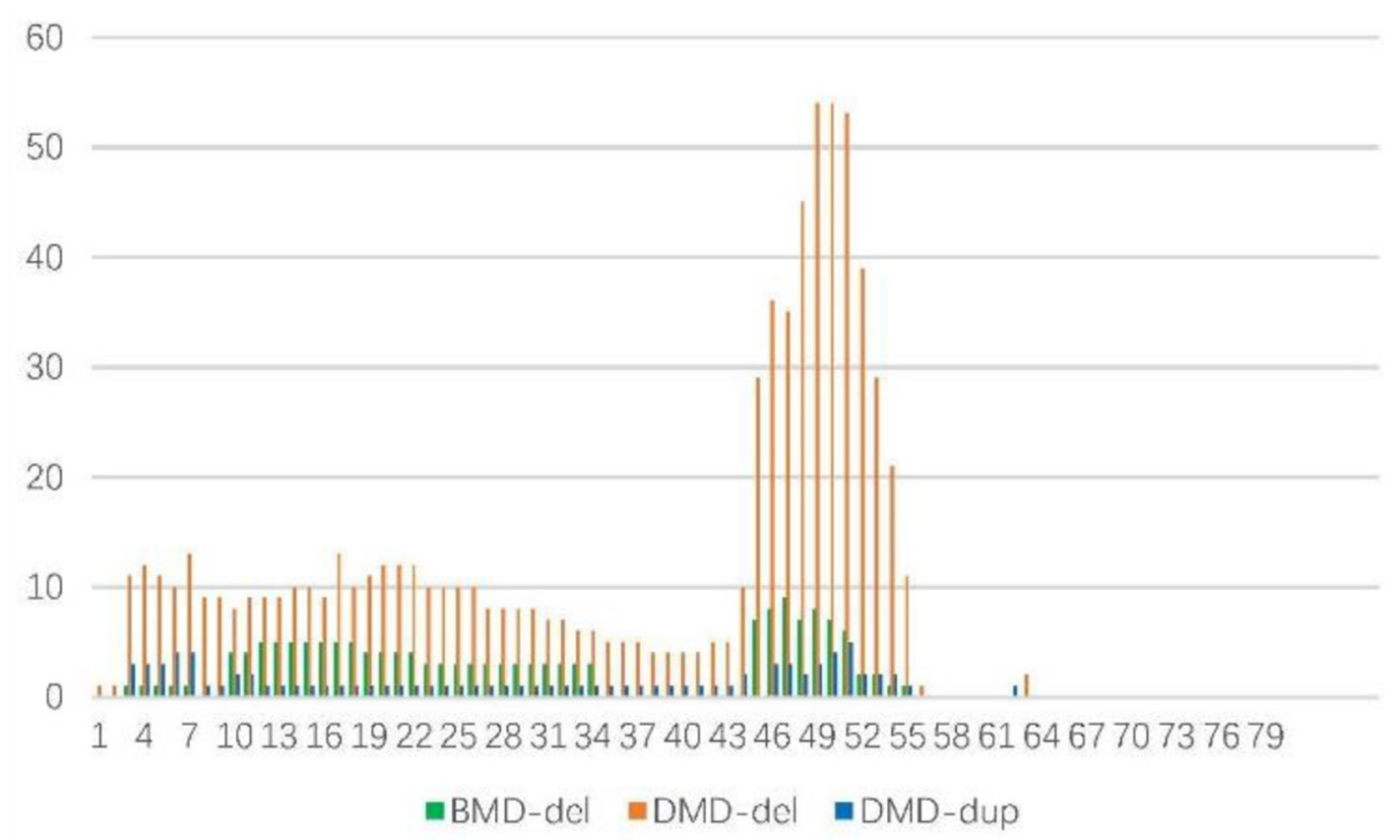
Frequency of deletions/duplications of DMD gene exons in 169 cases.

One hundred fifty three cases of deletions were as follows: those in a single exon was the most frequent (28.1%), followed by 5 (13.1%), 2 (9.8%), 3 (8.45%), 6 (7.19%), 8 (7.19%), and 7 (6.54%), Figure 3. Therefore, 106 cases (69.3%) had 6 or fewer exon deletions. Deletion of more than 10 exons accounted for only 22 cases (14.38%). In cases of BMD and DMD, there were 5 cases (29.41%) and 17 cases (12.5%) with deletion exons more than 10, respectively (χ^2^-test, *P* = 0.06). Figure 3 shows that exons 10–34 (60.1%) were most commonly missing in BMD, followed by 45–51 (32.9%). The most common exon deletion in DMD was 44–55 (54.1%), followed by exon 3–26 (32.38%). Sixteen cases with duplication were DMD, mainly exons 46–54 (30.59%) and 3–7 (20%), Figure 3.

### “Reading-frame rule” combined with “walking alone milestone” in prediction for DMD

In 169 cases, 12 cases of BMD are in the reading frame, and 132 cases of DMD are out of the reading frame, in which 85.2% were in agreement with the rule of reading-frame (86.8% for DMD and 70.59% for BMD). Sensitivity and specificity were 86.8 and 70.59%, respectively, assuming that DMD was the case out of the reading frame and BMD was the case in the reading frame. Sensitivity and specificity were 61.18 and 100%, respectively, assuming that the case was BMD when walking alone milestones were younger than 18 months. The case was DMD when the milestone was older than 18 months. The combined sensitivity and specificity were 96.05 and 70.59%, respectively. The specificity remained unchanged, sensitivity increased by 9.25%, and the diagnostic coincidence rate increased from 85.2 to 93.49% (χ^2^-test, *P* = 0.014; Table 3).

**Table 3:** “Reading-frame rule” and “walking alone milestone” in prediction for DMD.

	**Sensitivity (%)**	**Specificity (%)**	**Diagnostic coincidence rate (%)**	*P*-value^*^
Reading-Frame rule	86.8	70.59	85.2	–
Walking alone milestone	61.18	100	60.09	–
Reading-Frame rule and walking alone milestone	96.05	70.59	93.49	0.014

* *P-value: χ^2^-test for the total efficiency of joint prediction and the reading-frame prediction*.

## Discussion

### Predicting DMD as early as possible is the premise of timely treatment

Because DMD pathology is caused by the lack of functional dystrophin, restoring the function or expression of dystrophin is an apparent therapeutic approach. Loss of muscle tissue and function starts at an early age and is currently irreversible. Thus, although restoration of muscular dystrophy protein expression is called upon to slow or even stop the progression of DMD, it will not restore any muscle tissue that has been lost.

The standard way to confirm DMD/BMD recently included serum creatine kinase testing, genetic confirmation, and dynamic assessment of patient scores on motor ability tests (8). The most widely used treatment remains the use of corticosteroids and angiotensin-converting enzyme inhibitors to control the symptoms of cardiomyopathy and rehabilitation and symptomatic support to prolong patient life (1). Current therapies, such as exon 44/45/51/53 skipping and treating DMD with non-sense mutations, have been gradually applied to clinical practice (9–11). Even gene therapy, which leads to high expression of functional muscular dystrophy protein, is not expected to cure when given to patients who have lost most muscle tissue and function. Clemens and others found that DMD patients as young as 4 years exhibited improvements in dystrophin levels and timed motor tests following the 53 exons skipping treatment. Hence, they believed that these cases should be treated before 5 y (12). There are even studies using exon-skipping in patients up to 6 months old (ClinicalTrials identifier: NCT03218995). Pre-treating patients before gene therapy using an exon-skipping approach could potentiate the effect of gene therapy. Such pre-treatment would allow lower and safer vector doses to bring about a higher level of dystrophin expression in the long term (13–15).

Early intervention is essential, but early identification of DMD/BMD is the first step. Some indicators were applied in the early prediction of DMD, and the compound indicators were rarely reported. The reading-frame rule is one of the recognized DMD prediction indicators, and the joint prediction of the walking alone milestone can effectively improve the sensitivity and diagnostic coincidence rate.

Methods for predicting DMD/BMD early and predicting DMD severity developed. CK is a sensitive biomarker because elevated blood levels (10 and 100 times higher than the upper limit) indicate severe muscle damage (16). CK is rather unspecific because plasma levels are also elevated in many forms of other muscle injury and are influenced by other factors, such as muscle mass, age, and muscle activity. Genotype (including reading-frame), modified genes, muscle-specific microRNA, and developmental milestones have been studied to predict DMD/BMD and DMD severity.

In 1988, Monaco et al. proposed the reading-frame rule, and related research supports this hypothesis (17). In the study of Aartsma-Rus, 91% agreed with this rule (3). It had been reported that the reading-frame rule held in 90% of DMD and 94% of BMD cases (18). Later studies suggested that BMD might result in exceptions to the reading frame rule in more cases, perhaps up to 30% (19, 20). However, our study found that the positive prediction rate of the reading-frame rule was 85.2% for all cases, 86.8% for DMD, and 70.59% for BMD. Early clinical treatment is highly urgent for DMD cases, but 12.6% cannot be diagnosed early. Although the reading frame helps predict the severity of skeletal muscle weakness, there is still some phenotypic variability within the prediction.

Some authors reviewed 4000 patients and found that some cases in the reading-frame had DMD phenotype, which was more likely to be in the in-frame deletions starting and/or ending at the extreme ends of the protein (21). In our cases, 20 in-frame deletion/duplication cases had DMD phenotype, as shown in Figure 2. We did not find this feature, which may be related to the small number of our cases. The peak locations for BMD exon deletion are exons 45–51 and 3–26, similar to DMD, exons 44–55 and 3–26. Our study found no difference in the number of exon deletions between DMD and BMD. It is not practical to judge DMD/BMD by exon deletion/duplication site. In cases of BMD and DMD, there were 5 cases (29.41%) and 17 cases (12.5%) with deletion exons more than 10, respectively (χ^2^-test, *P* = 0.06). In the study of scholar Juan Yang, among 118 cases of exon duplication of DMD gene in the Chinese population, there were 9 cases of BMD, indicating that exon duplication is more likely to occur in DMD cases (22). No exon duplication was found in our BMD cases, which may be related to the small number of cases in us.

Early gross motor development milestone delay is a clinical characteristic of DMD, but is not necessary for BMD (23). van Dommelen found that between 12 and 36 months of age, differences in the attainment of developmental milestones concerning gross motor activity increased with age (7). Sitting, crawling, and walking alone were considered important milestones in motor development. The most significant lag in DMD is walking alone, which is related to DMD most easily involving the lower limbs (6). Therefore, it is appropriate for us to take the age of first walking to represent the backward development of gross motor. In our study, the walking alone milestone was 12.88 ± 0.61(month) in BMD group, and 18.03 ± 7.12 (month) in DMD group. In DMD cases, 93 cases (61.18%) had an independent walking delay. We believe that the walking alone milestone delay has a limited predictive effect on DMD.

### The combined prediction of the reading-frame rule and walking alone milestone has the potential for early diagnosis of DMD

Therefore, we combined the reading-frame rule and gross motor milestone for the above reasons. Our results revealed that combined variables improved the prediction efficiency compared to the isolated reading-frame rule. So, for the first time, we proposed the integration of the two variables to predict DMD. Furthermore, both the genotype and walking alone milestones are very constant and stable, easy for doctors to obtain. Because of the low cost and no harm to patients, it is conducive to promotion. In our study, the reading-frame rule combined with the walking alone milestone increased the diagnostic coincidence rate of DMD from 85.2 to 93.49%. The sensitivity increased significantly, but the specificity did not decrease. Therefore, we believe that the combined index of the above two variables might have application potential for early DMD prediction.

### Study strengths and limitations

Our study is a retrospective study, and more detailed motor milestones, early fine motor and assessment of intellectual development level are not available, limiting our prediction age in DMD. We hope to carry out prospective studies in the future. The severity of BMD and DMD is different, and the treatment is different, so early prediction is of great significance. The severity of DMD varies, with some patients living in wheelchairs before the age of 10 and some losing their independent ambulation at the age of 15. Therefore, progress in practical DMD prediction tools is expected to stratify patients accurately and timely.

## Conclusion

The reading-frame rule is widely used in DMD prediction. However, its prediction efficiency still needs to be improved. The motor development milestone delay, especially when they are 18 months still cannot walk alone, could predict a considerable part of DMD (7, 24, 25). But the specificity is poor. Our study proposed that the combined prediction of the above two indicators significantly improved the early diagnosis rate of DMD and provided a new tool for earlier diagnosis. Because these two indicators are stable, easy to obtain, and have the potential to be widely promoted in the future. Prospective studies with large samples and multiple regions are still needed to verify it further.

## Data availability statement

The original contributions presented in the study are included in the article/supplementary material, further inquiries can be directed to the corresponding author.

## Author contributions

Y-lM and X-yC contributed to conception and design of the study. G-hC, L-fS, YuW, R-lY, and YiW organized the database. W-hZ performed the statistical analysis. Y-lM and W-hZ wrote the first draft of the manuscript. All authors contributed to manuscript revision, read, and approved the submitted version.

## Funding

This research was supported by the key science and technology project of Henan provincial department of science and technology (project nos. 222102310705 and 212102310446).

## Conflict of interest

The authors declare that the research was conducted in the absence of any commercial or financial relationships that could be construed as a potential conflict of interest.

## Publisher's note

All claims expressed in this article are solely those of the authors and do not necessarily represent those of their affiliated organizations, or those of the publisher, the editors and the reviewers. Any product that may be evaluated in this article, or claim that may be made by its manufacturer, is not guaranteed or endorsed by the publisher.
